# The Electrical, Mechanical and Surface Properties of Thermoplastic Polyester Elastomer Modified by Electron Beta Radiation

**DOI:** 10.3390/polym10101057

**Published:** 2018-09-22

**Authors:** David Manas, Ales Mizera, Milan Navratil, Miroslav Manas, Martin Ovsik, Stanislav Sehnalek, Pavel Stoklasek

**Affiliations:** 1 Faculty of Applied Informatics, Tomas Bata University in Zlin, CEBIA-Tech, Nad Stranemi 4511, 760 05 Zlin, Czech Republic; manas@utb.cz (D.M.); navratil@utb.cz (M.N.); manas@fai.utb.cz (M.M.); sehnalek@fai.utb.cz (S.S.); pstoklasek@utb.cz (P.S.); 2 Faculty of Technology, Tomas Bata University in Zlin, Vavreckova 275, 760 01 Zlin, Czech Republic; ovsik@utb.cz

**Keywords:** thermoplastic polyester elastomer, irradiation, radiation cross-linking, electrical and mechanical properties

## Abstract

The main advantages of Thermoplastic Polyester Elastomers (TPE-E) are their elastomer properties as well as their ability to be processed in the same way as thermoplastic polymers (e.g., injection moulding, compression moulding and extrusion). However, TPE-Es’ properties, mainly their mechanical properties and thermal characteristics, are not as good as those of elastomers. Because of this TPE-Es are often modified with the aim of improving their properties and extending their range of application. Radiation cross-linking using accelerated electron beams is one of the most effective ways to change virgin polymers’ properties significantly. Their electrical (that is to say permittivity and resistivity measurements), mechanical (that is, tensile and impact tensile tests), as well as surface (that is, nano-indentation) properties were measured on modified/cross-linked TPE-E specimens with and/or without a cross-linking agent at irradiation doses of 0, 33, 66, 99, 132, 165 and 198 kGy. The data acquired from these procedures show significant changes in the measured properties. The results of this study allow the possibility of determining the proper processing parameters and irradiation doses for the production of TPE-E products which leads to the enlargement of their application in practice.

## 1. Introduction

TPE-E is a member of a relatively new group of thermoplastic elastomers whose production grows year on year, mainly due to their price and excellent properties. They are often used as a substitute for classic elastomers. However, the TPE-E properties do not attain the levels of elastomer properties over the entire temperature range, which leads to some limitations. Because of this granulate manufacturers prepare TPE-E with precise properties for the needs of the automotive and electrical industries [1,2]. It is a block copolymer which typically consists of hard segments composed of several short chain esters, (e.g., tetramethylene terephthalate), and soft parts containing aliphatic polyether and polyester glycol [3]. Nagai et al., at the end of the 1990s, studied the influence of weather on TPE-E and evaluated the impact of photo-degradation, thermal degradation and hydrolysis on this material. As with other thermoplastic elastomers the hard-crystalline domains of TPE-E ensure elastomer properties and heat resistance while soft amorphous ones have good low-temperature flexibility [4,5].

Several possibilities exist regarding ways to improve mechanical and electrical properties; in particular temperature stability for example, by using inorganic particles (which is a common practice). Nowadays, micro-sized or nano-sized particles, (e.g., clay, talc mica, silica, fly ash, carbon nanotubes, etc.), are used as filler that improves certain TPE-E properties [6,7,8,9,10,11,12]. The resulting properties depend on the filler used and its concentration and shape can provide better cohesion between the matrix and the filler. The larger the specific surface area of the filler the greater the cohesion with the matrix. Similarly, the particle size and dispersity of the TPE-E particles are manifested in the resulting properties [13,14]. Dielectric properties can be improved by adding nanoclay, on the other hand the conductivity can be improved by adding a metallic filler. The amount of filler is limited by the workability of the resulting mixture where the optimal ratio between the filler and the matrix has to be found so that the utility properties and the economics of the process are optimal [15,16,17].

Other possibilities for improving TPE-E properties and temperature stability especially for instance, can be the use of radiation cross-linking using electron beta rays. This can effectively be used to modify certain properties according to specific requirements. This type of modification has been widely known for a long time now and is widely used in the manufacturing of cables and hoses. Some polymers can be cross-linked without the help of cross-linking agents, (e.g., TPE-E, LDPE, and HDPE); others need a cross-linking agent, (e.g., polyamides and polypropylenes), without which this predominantly leads to degradation. A cross-linking agent can also be used for thermoplastics that are mainly cross-linked. It is mainly used to reduce the irradiation dose and to accelerate the cross-linking process. Cross-linked thermoplastics lose the ability to re-melt which greatly deteriorates the possibilities of their processing after the end of their life cycle or the processing of production waste [18,19,20,21,22].

Many studies deal with the measurement and evaluation of the mechanical properties of variously modified TPE-E; however, the measurement of their electrical properties is an area which has yet to be fully explored, and especially, about radiated cross-linked TPE-E [23,24,25,26]. Our previous study compared the mechanical properties of TPE-E, with, and without, a cross-linking agent. It was determined that for mechanical properties the addition of a cross-linking agent at low doses, (up to 66 kGy) does not affect it. On the other hand, at higher doses (up to 198 kGy) the effect of the cross-linking agent on the mechanical behaviour of TPE-E was significant [27].

The main aim of this study is to obtain as much information about the possibilities of exploiting cross-linking agents in the TPE-E structure in comparison to a material without cross-linking agents for the TPE-E behaviour at different irradiation doses. The acquired information from electrical and mechanical measurements including the surface properties of the TPE-E material being studied, leads to a comprehensive set of knowledge/information that will provide not only scientists but polymer materials manufacturers and associated engineers with lots of room for further improvements/extensions of TPE-E application possibilities.

## 2. Materials and Methods

### 2.1. Materials

A thermoplastic polyester elastomer (Teknor Apex, Pawtucket, RI, USA) without (TPE-E, PTS-UNIFLEX-E25D/M*M800 natural) and with (V-TPE-E, V-PTS-UNIFLEX-E25D/M*M800/20 natural) a cross-linking agent was used as the basic polymer materials. After the radiation cross-linking by beta-electron rays, these materials change the structure from the thermoplastic material to not re-meltable one. For easier orientation in the text, the cross-linked TPE-E and V-TPE-E are marked TPE-Ex and V-TPE-Ex, respectively.

### 2.2. Specimen Preparation

**Figure 1:**
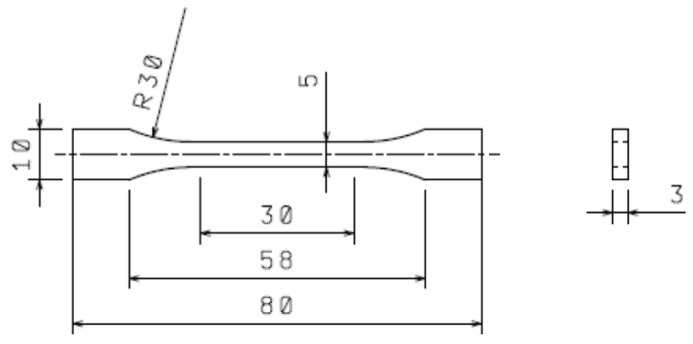
Dimensions of testing specimen.

An ARBURG Allrounder 170U 150-30 injection moulding machine (Arburg, Loßburg, Germany) was used for specimen preparation, with the processing conditions to comply with the TPE-E and V-TPE-E producer’s recommendations (Table 1). For all mechanical measurements, the samples of 1BA type produced according to ISO 527-2 standard [28] were used. The dimensions and shape of test specimens are apparent from Figure 1.

100 × 100 × 0.3 mm sheets were used to measure the electrical properties. They were produced by the compression moulding method, at a temperature of 200 ${}^{\circ }$C, and a pressure of 5 MPa. The thickness of the film was measured with a micrometre, in 15 locations and ranged within a tolerance range of ±0.02 mm, which is appropriate for accurate measurements and their subsequent evaluation of resistivity and permittivity.

Irradiation of tested TPE-E and V-TPE-E was performed with the kind help of BGS Germany, in the BGS Wiehl plant using accelerated electrons. For these purposes, RHODOTRON E-beam accelerator (IBA, Tongeren, Belgium) with an energy of electrons 10 MeV was used. Irradiation process of TPE-E and V-TPE-E specimens carried out at general conditions (air atmosphere, ambient temperature 23 ${}^{\circ }$C) as it is performed in engineering practice. The range of the doses was determined by the experience from the practice of the industrial irradiation in the range from 33 to 198 kGy. Each passage under the accelerator scanner is equal to 33 kGy. The required dose was determined according to accelerator parameters, and its correctness was measured by the dosimeter. Nylon FWT 60-00 dosimeter (Far West Technology, Inc., Goleta, CA, USA) was used to check the correct radiation dose, following analysis was carried out on spectrophotometer Genesis 5, according to the ASTM 51261 standard [29]. Required and real surface value of gain irradiation dose is described in Table 2.

### 2.3. Gel Content

Gel test is done to find the content of non-dissolved phase-gel of the given material according to standard ASTM D 2765-test method C [30]. The portion of 0.5 g (of the electron beam irradiated TPE-Ex and V-TPE-Ex material) weighed with a precision of five decimal places on weighing apparatus “SWISS MADE EP 125 SM” was mixed with 100 mL of solvent. Xylene was used for TPE-Ex and V-TPE-Ex because it dissolves, on the other hand the cross-linking part does not dissolve. The mixture was extracted for 24 h. Then solutes were separated by distillation. After removing the residual xylene, the cross-linked extract was dried for eight hours in vacuum at 100 ${}^{\circ }$C. The dried and cooled residue was weighed again with precision to five decimal places and compared to the original weight of the portion. The result is stated in percentage as the degree of cross-linking.

(1) $$G_{i}\;=\;\frac{m_{3}-m_{1}}{m_{2}-m_{1}}\;100$$

where $G_{i}$ is the degree of cross-linking of each specimen expressed in percentage, $m_{1}$ is the weight of the cage and lid in milligrams, $m_{2}$ is the total of weights of the original specimen, cage and lid in milligrams, and $m_{3}$ is the total of the weights of the residue of specimen, cage and lid in milligrams.

### 2.4. Volume Resistivity Measurement

Volume resistivity $\rho_{v}$ is defined as the electrical resistance through a cube of insulating material. The standard resistivity test method for an insulator involves applying a voltage step using voltage source, for a specified period. In our case, the voltage-current force measurement technique is used. The measuring system consists of a precise electrometer a Keithley 6517A with an 8009 resistivity test fixture. The sample is positioned between the electrodes and a voltage of 500 V is applied. The time dependence of the resultant current is measured for 60 seconds, due to polarising procedures; after this period the current flow is sufficiently stabilised. The sample resistance and volume resistivity are calculated according to Ohm’s Law and the geometrical considerations of electrodes used, as follows:

(2) $$\rho_{v}=\frac{A}{t}\frac{U}{I}$$

where $\rho_{v}$ is volume resistivity [Ωm]; *A* is the effective area of the guarded electrode for the particular electrode for the arrangement employed [m${}^{2}$]; *t* is the sample thickness [m]; *R* is the calculated resistance [Ω]; *U* is voltage [V]; and I is current [A].

The above-mentioned test procedure conforms to ASTM D-257 [31] and IEC 62631-3-1 [32] Standards for DC Resistance of Insulating Materials.

### 2.5. Permittivity Measurement

Loss factor tan(δ) and relative permittivity $\epsilon_{R}$ are two important material parameters that enable one to evaluate substances (insulators), which are evaluated among themselves. Based on these parameters, it is possible to determine the exact application of the materials used. The loss factor characterises insulators in dielectric loss terms. Relative permittivity corresponds to electrostatic forces in materials and is defined as the permittivity of a given material, relative to the permittivity of a vacuum. The parallel-plate capacitor method was chosen for this measurement procedure. It involves sandwiching a thin sheet of material between two electrodes, to form a capacitor. The measuring system consists of a Keysight E4980A precision LCR meter, and the HP 16451B dielectric test fixture. After initial calibration the capacitance value, along with a parallel-equivalent circuit model and the loss factor of the material, is measured at frequency ranges from 20 Hz to 1 kHz. The values obtained, and the geometrical considerations of the electrodes used, as well as relative permittivity are calculated as follows:

(3) $$\varepsilon_{R}\left(\omega \right)=\frac{t\;C_{p}\;\left(\omega \right)}{\varepsilon_{0}\;A}$$

where ω is angular frequency [rad s${}^{-1}$]; t is the thickness of the sample [m]; Cp is the capacitance value [F], $\varepsilon_{0}$ is the permittivity of the vacuum [Fm${}^{-1}$]; and *A* is the effective area of the guarded electrode [m${}^{2}$]. The dielectric properties’ results presented herein are given for 1 kHz frequency since it is usually provided in technical standards.

### 2.6. Tensile Test

The tensile test was carried out on a T 2000 Alpha Technologies testing machine at ambient temperature 23 ${}^{\circ }$C according to ISO 37 standard [33], under a constant speed of elongation of 500 mm min${}^{-1}$. The used testing samples were in the shape of a shovel as displayed in Figure 1. 15 samples were tested, and their ultimate tensile strength and elongation at break values were evaluated in TestExpert II, MS Excel and MiniTab programs. In all figures arithmetic mean and standard deviation are used.

### 2.7. Impact Tensile Test

The tensile impact test was carried out on Zwick HIT50P equipment (Zwick, Ulm, Germany) at ambient temperature of 23 ${}^{\circ }$C according to standard ISO 8256 [34]. 50 J impact hammer was used on this test. 15 specimens (Figure 1) were tested, and their ultimate impact tensile strength values were evaluated in TestExpert II, MS Excel and MiniTab programs. Arithmetic mean and standard deviation were used as the statistical parameters in this measurement.

### 2.8. Nano-Indentation Test

Nano-indentation test was performed using a nano-indentation tester (nano hardness tester), Anton Paar (Graz, Austria) according to the ISO 14577 standard [35]. The Depth-Sensing Indentation (DSI) method used enables measuring of the force acting on the Vickers indentor (Anton Paar, Graz, Austria) which is made of diamond, the shape of a cube corner and the displacement of the indentor during the test. In the present study, the maximum load used was 0.1 N and loading and unloading rate was 0.2 N min${}^{-1}$, while the holding time was 90 s.

Measurements of all properties mentioned above were performed 30 times to ensure statistical correctness.

## 3. Results and Discussion

### 3.1. Gel Content Determination

Table 3 shows the gel content values as measured using the xylene extraction method, according to ASTM D 2765, test method C [30]. At the lowest irradiation dose of 33 kGy no gel content was measured for both types of TPE-Ex and V-TPE-Ex by this method. As with irradiated TPE-Ex with a dose of 66 kGy the gel content was not measured; the possible cause may be the formation of micro-gels that were not entrapped by the screen during extraction. On the other hand, the gel content of V-TPE-Ex increased by 78% at this dose. For TPE-Ex a gel content of 57% was measured at a dose of 99 kGy. With increasing doses of radiation the gel content of the both tested TPE-Ex and V-TPE-Ex gradually increased. At the maximum irradiation dose of 198 kGy the gel content difference between TPE-Ex and V-TPE-Ex is 19%. From the measured values it can be concluded that the presence of a cross-linking agent in the TPE-E structure has a significant effect on the reduction of the radiation dose, which allows for greater production results and reductions in production costs.

### 3.2. Volume Resistivity Measurement

The first quantity measured was volume resistivity which serves as an indicator of the electric properties; the higher this is the better the insulation properties of the material. Since TPE-E belongs to a group of materials with excellent electric properties a large change in these properties is not expected, due to the addition of a cross-linking agent and subsequent cross-linking with beta-electron radiation.

**Figure 2:**
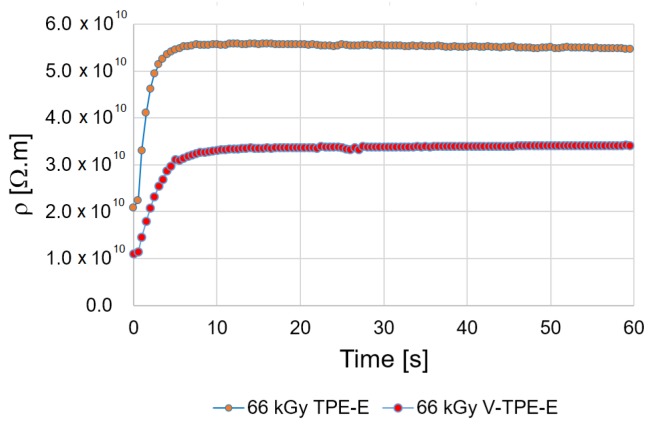
Measurement course of the volume resistivity at voltage of 500 V.

As can be seen in Figure 2 the volume resistivity measured over the period of sixty seconds seems primarily to increase exponentially and then the value becomes reliably stable. For comparison purposes the electrification time of 60 s was chosen, with an applied voltage of 500 V.

**Figure 3:**
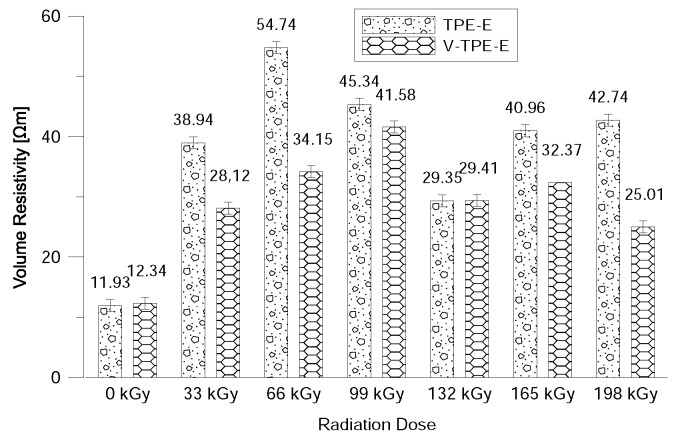
Volume resistivity at voltage of 500 V after 60 s.

The value of the volume resistivity values was measured in the order of dozens of GΩ; while the measurement error proved relatively small as shown in Figure 3. However, it can be observed from the measured values that radiation cross-linking does not impair the electric properties; on the contrary; there is a slight improvement. For V-TPE-Ex there was a slight decrease in volume resistivity than for TPE-Ex except for the irradiation dose of 132 kGy, despite this drop in values being higher than virgin TPE-E.

### 3.3. Permittivity Measurement

**Figure 4:**
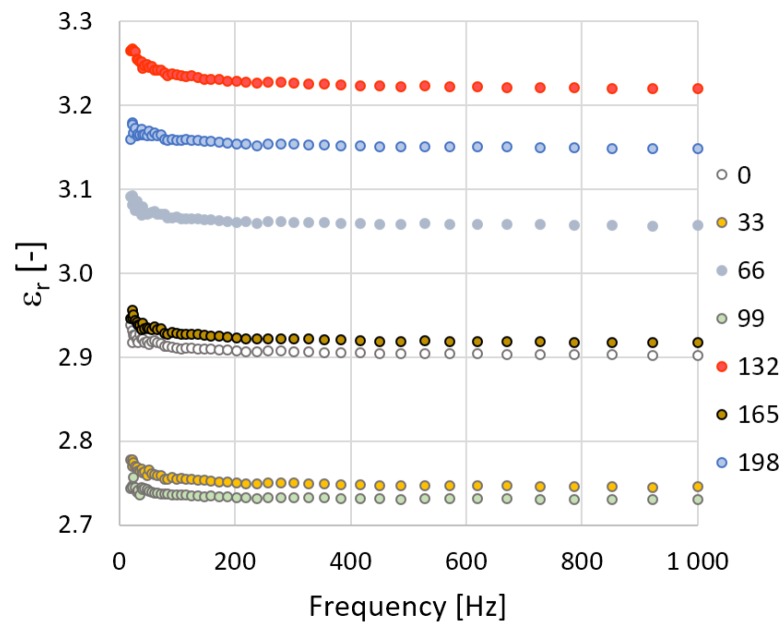
TPE-E—measurement course of the relative permittivity in dependence on frequency.

**Figure 5:**
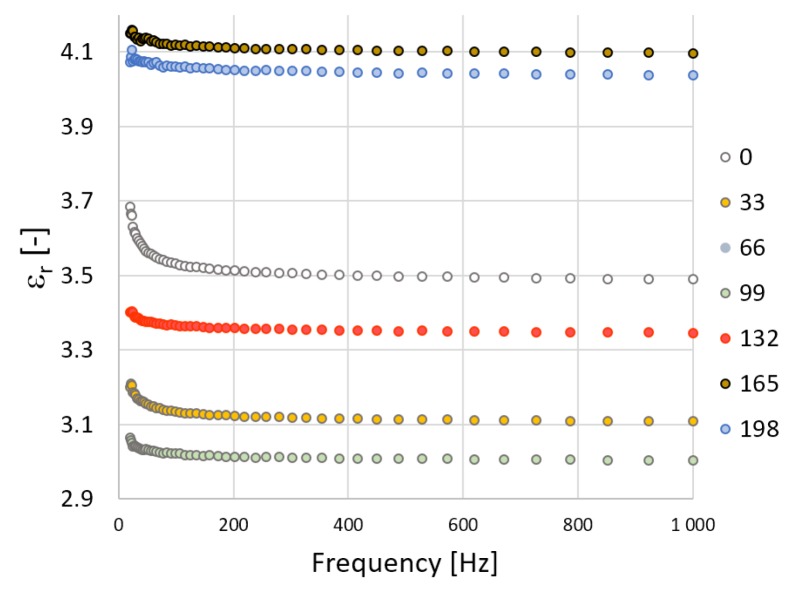
V-TPE-E—measurement course of the relative permittivity in dependence on frequency.

**Figure 6:**
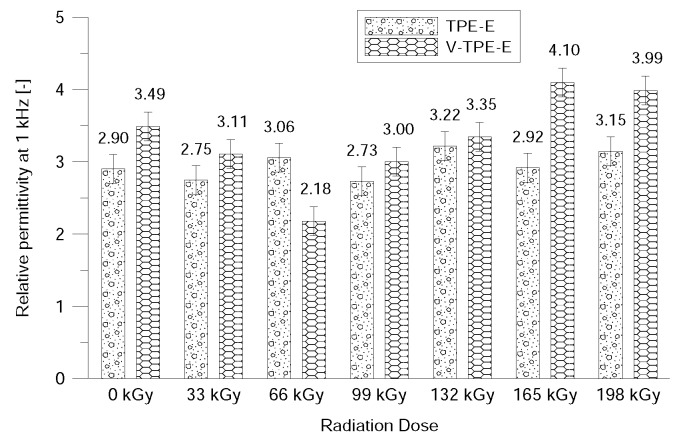
Relative permittivity measured at 1 kHz.

Further measurements of the electrical parameters included the relative permittivity and the loss factor, which also served for the evaluation of the insulation characteristics of the materials. Concerning the fact that TPE-E is non-polar dielectric both relative permittivity and the loss factor are not dependent on frequency or temperature. Figure 4 and Figure 5 show the frequency dependence of relative permittivity which is almost constant except for at lower frequencies. This is caused due to uncertainties relating to the measurement method for the lowest frequency range. The radiation dose does not affect the relative permittivity of TPE-Ex; however, with V-TPE-Ex the radiation dose increased to 66 kGy and led to a slight decrease and with a further increase in the radiation dose, there was a gradual increase of up to 165 kGy, where the relative permittivity value stabilised at roughly about 4. It can also be observed from the relative permeability measurements that TPE-E irradiation did not result in any radical deterioration of the dielectric properties (Figure 6).

**Figure 7:**
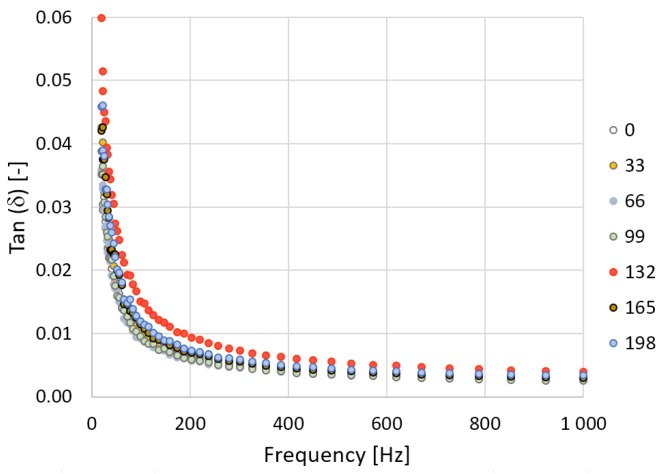
TPE-E—measurement course of the loss factor in dependence on frequency.

**Figure 8:**
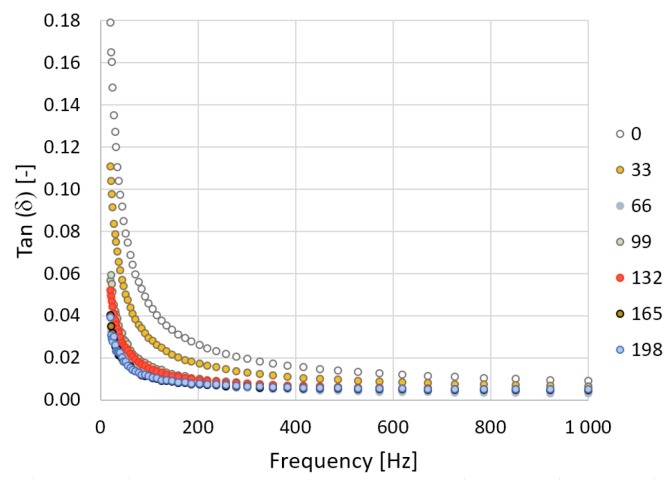
V-TPE-E—measurement course of the loss factor in dependence on frequency.

**Figure 9:**
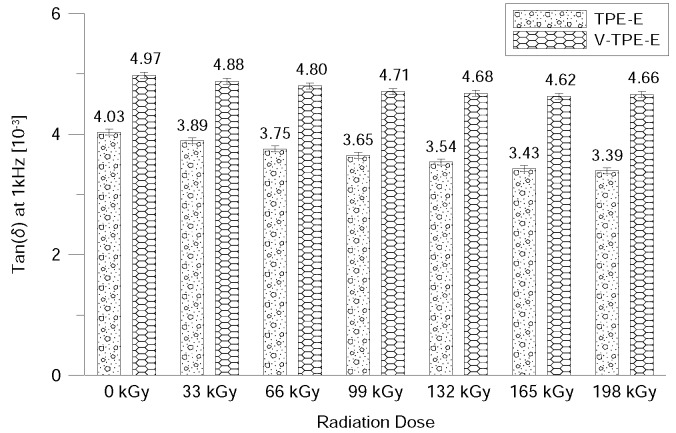
Loss factor measured at 1 kHz.

In Figure 7 and Figure 8 it can be seen that the loss factor decreases exponentially over the frequency. The loss factor decreases in line with higher irradiation doses. This also means there are decreases in electric conductivity values. The addition of cross-linking agents to the V-TPE-E led to the growth of the loss factor value by 0.001, within the overall range of the radiation doses. This may mean slightly greater heating of the material in comparison to TPE-E under the influence of the through-flow of the electric current (Figure 9).

### 3.4. Tensile Tests

Following the electrical properties’ tests further tests were made on the mechanical properties and in particular the static and impact tensile properties were measured. Regarding the static tensile properties, the ultimate tensile strength and elongation at break were evaluated. In case of the impact tensile properties the ultimate impact tensile strength was evaluated; elongation at break was not measured due to overloading the test specimens with a hammer energy of 50 J.

**Figure 10:**
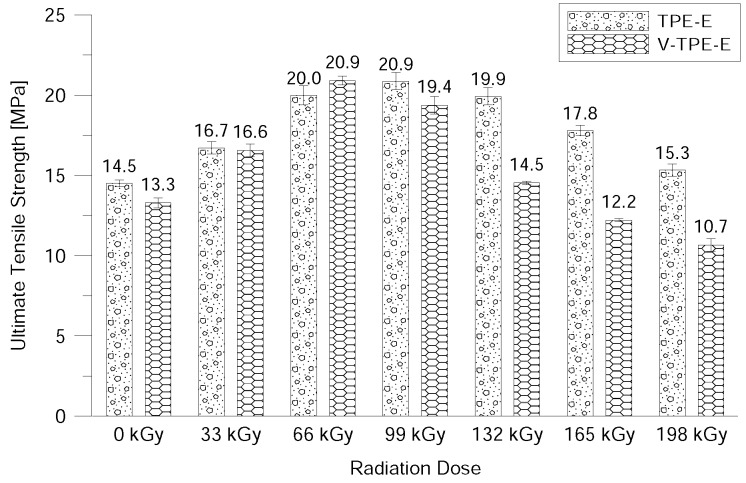
Ultimate tensile strength dependence on the radiation dose.

The ultimate tensile strength increases gradually at low radiation doses (up to 66 kGy) while the effect of the cross-linking agent is minimal. With an increasing radiation dose (up to 198 kGy) the ultimate tensile strength drops. V-TPE-Ex at 198 kGy is about 4.6 MPa lower than the value of TPE-Ex at the same radiation dose (Figure 10). Decreases in ultimate tensile strength indicate the gradual degradation of the material due to the amount of radiation. From this point-of-view the optimal dose would appear to be between 66 and 99 kGy where the highest ultimate tensile strength was measured and correlated with gel content measurements showing the jump increase in gel content that was measured at these doses.

**Figure 11:**
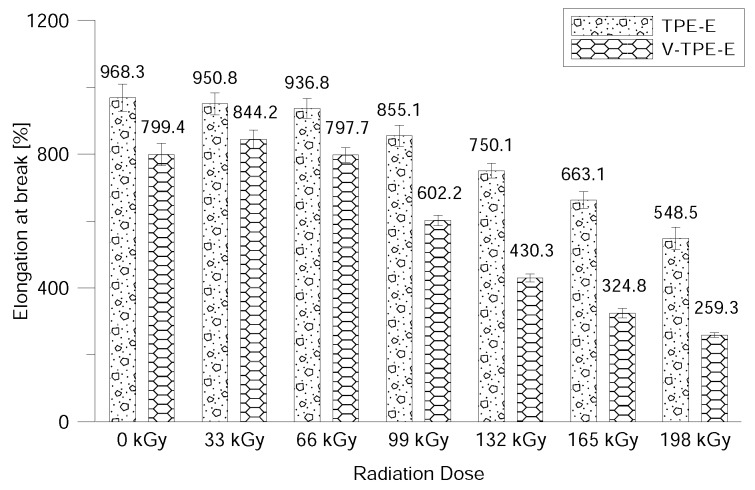
Elongation at break dependence on the radiation dose.

Figure 11 depicts the dependence of elongation at the break on the radiation dose. Based on this measurement process it can be observed that cross-linking agents in V-TPE-E worsen elongation. Only by the fact of their being present; the ensuing radiation cross-linking is the value of elongation at break and is constant in samples up to a radiation dose of 66 kGy; this then drops sharply to 259.3% at a dose of 198 kGy. A similar pattern is also observed for TPE-E; nevertheless, the elongation at break value is, at 198 kGy, more than double that of V-TPE-Ex. At a dose of 66 kGy the difference in elongation at break between TPE-Ex and V-TPE-Ex is 139.1%. The decline in elongation at break increases with radiation doses. This is probably caused on the one hand, by the “cramping” of the cross-linking networks with the “stiffness” of cross-linking; which is no longer as resilient as virgin TPE-E. On the other hand high doses lead to the degradation which also could contribute to elongation changes.

**Figure 12:**
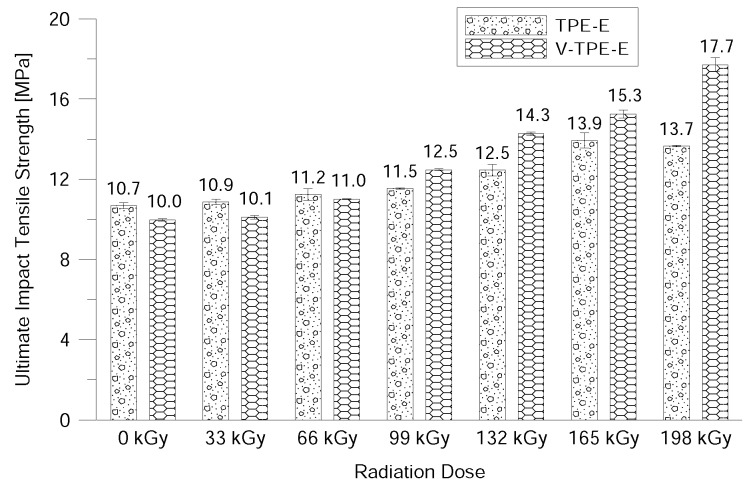
Ultimate impact tensile strength dependence on the radiation dose.

Figure 12 shows the dependence of the maximum impact tensile strength on the radiation dose. Unlike the tensile properties measured under static load, the impact load led to the opposite trend, with the ultimate impact tensile strength gradually increasing to a maximum irradiance of 198 kGy. The material does not have time to adapt to the applied load and thus fails to capture changes taking place in the material sensitively enough. However, it can be noted that the addition of a cross-linking agent does not worsen the tensile properties at impact loads even at higher radiation doses; on the contrary, the ultimate impact tensile strength increased by 7.74 MPa for V-TPE-Ex at 198 kGy as compared to V-TPE-E that was not irradiated. Here, the curves correlate very well with the gel content measurements.

### 3.5. Nano-Indentation Test

The surface properties were evaluated using DSI—to be more precise the nano-indentation properties (i.e., indentation hardness, indentation modulus, elastic deformation work and plastic deformation work) after exposure to beta-electron radiation.

**Figure 13:**
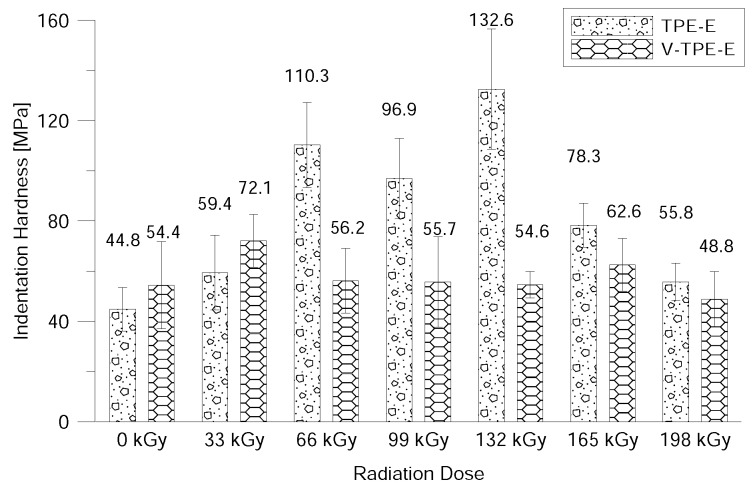
Nano-indentation hardness dependence on the radiation dose.

From Figure 13 it is apparent that the indentation hardness of V-TPE-Ex lies to the full extent within the range of radiation doses (except of the dose of 33 kGy) used in the measurement error area where the indentation hardness is around 54 MPa and it is in all cases oscilating around the value of indentation hardness of non-irradiated V-TPE-E. On the other hand TPE-Ex showed an increase in indentation hardness in the radiation dose ranging from 33 to 132 kGy with the maximum at the dose of irradiation of 132 kGy. This value is about 196 % higher than that of virgin TPE-E. Increases may have been influenced by the cross-linking of the surface layer; with further increases in radiation dose the indentation hardness dropped to the original virgin TPE-E value which could have caused the subsequent degradation of the surface layer.

**Figure 14:**
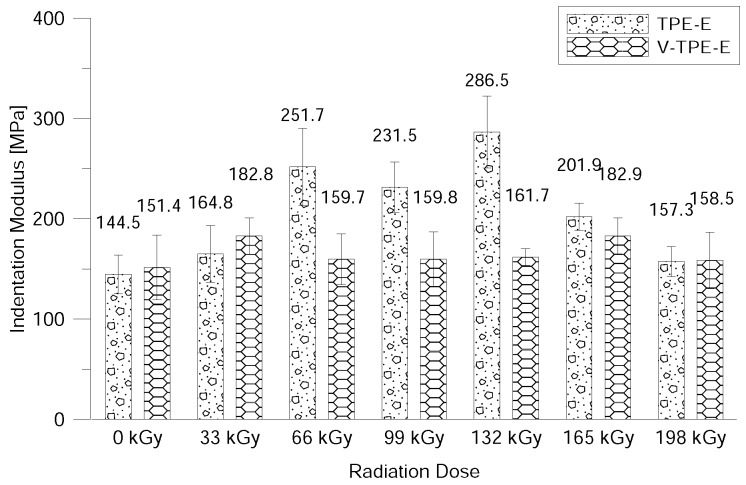
Nano-indentation modulus dependence on the radiation dose.

A similar tendency can be observed for the nano-indentation modulus results. The nano-indentation module V-TPE-Ex results fluctuate within the framework of measurement errors around 160 MPa in the whole range of the radiation dose. As regards TPE-Ex there is a significant growth in the measured values with its maximum attained with a radiation dose of 132 kGy and with the subsequent decrease in value to almost that of non-irradiated TPE-E (see Figure 14).

**Figure 15:**
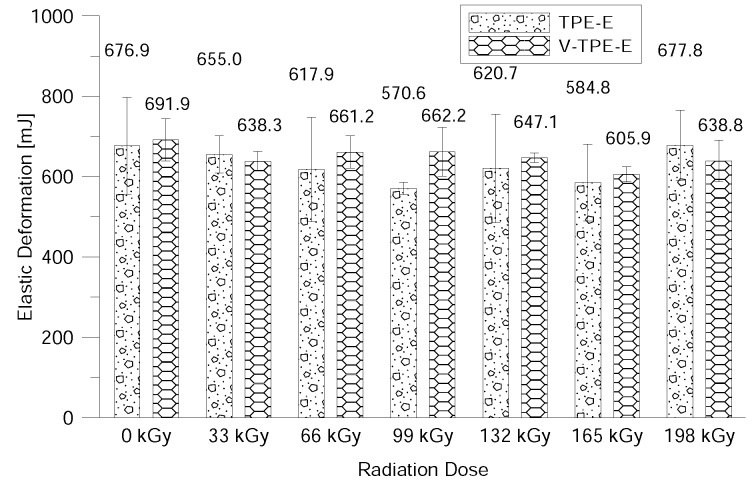
Elastic indentation work dependence on the radiation dose.

**Figure 16:**
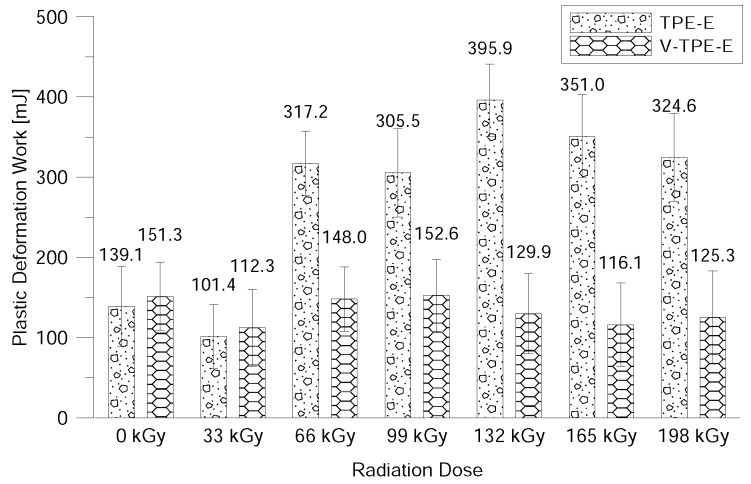
Plastic indentation work dependence on the radiation dose.

A similar tendency as when measuring the nano-indentation hardness and the modulus demonstrates the elastic and plastic deformation work (see Figure 15 and Figure 16).

## 4. Conclusions

Thermoplastic polyester elastomers are widely used due to their workability just like for thermoplastics, (injection moulding, extrusion, etc.), while they do achieve the properties of elastomers. The greatest limiting factor for TPE-E is its lower service temperature in comparison with elastomers. However, there are certain ways of modification, such as radiation cross-linking, which lead to increases of temperature stability [27,36]. TPE-E is generally a cross-linked material, (it does not require a cross-linking agent for cross-linking purposes); nevertheless, a 5% cross-linking agent is generally added, which ensures a faster reaction and maintains the stability of the cross-linking process. The choice of input material (with or without a cross-linking agent), depends on the resultant properties of radiation cross-linked TPE-Ex products.

From the gel content measurements, it is evident that V-TPE-Ex is cross-linked faster (at 66 kGy) than TPE-Ex (99 kGy). Similarly, the gel content of V-TPE-Ex at the maximum radiation dose (198 kGy) is much higher (93%), than for TPE-Ex (74%). Based on this measurement, the minimum radiation dose that is suitable for the radiation cross-linking of TPE-E can be determined. However, the specified minimum dose of radiation need not have the best results for electrical, mechanical and surface properties. It is necessary to optimise the radiation dose according to the final use of the product.

Based on electrical properties—volume resistivity, relative permittivity and loss factor, it was ascertained that radiation cross-linking does not worsen these properties, but on the contrary, certain radiation doses improve electrical properties. The addition of a cross-linking agent to V-TPE-E causes an increase in loss factor by 0.001 over the full range of radiation doses, which can cause slightly higher heating of the electric current than for TPE-E.

The tensile properties correlate with the gel content measurements while the optimal dose for V-TPE-Ex is 66 kGy, and for TPE-Ex is 99 kGy. These measured values indicate that, by adding a cross-linking agent a radiation dose reduction of 99 to 66 kGy could be achieved with the similar mechanical properties.

The last measurement in this study was the measurement of surface properties by using the nano-indentation method. This relates to the measurement of the hardness of the surface layer ranging from several units and up to tens of micrometres. This method can be used to evaluate the indentation hardness and modulus, the elastic and the plastic deformation work. With V-TPE-E, the cross-linking agent did not lead to changes in the surface properties throughout the whole range of radiation doses. On the other hand, TPE-Ex ranged from 66 and 132 kGy led to increases in indentation hardness and modulus.

Addition of a cross-linking agent to TPE-E does not lead to the impairment of the properties studied; in certain cases, improvements occurred. When is using a cross-linking agent the radiation dose can be reduced to 66 kGy which results in a certain reduction in production costs.

## Figures and Tables

**Table 1:** Injection moulding parameters.

**Arburg Allrounder 170U 150-30**		
Injection Velocity	50	mm s${}^{-1}$
Injection Pressure	45	MPa
Cooling Time	15	s
Mould Temperature	30	${}^{\circ }$C
Holding Pressure	40	MPa
**Temperature of Plasticizing Unit Zones**		
Temperature under the Hopper	30	${}^{\circ }$C
Temperature Zone 1	170	${}^{\circ }$C
Temperature Zone 2	185	${}^{\circ }$C
Temperature Zone 3	200	${}^{\circ }$C
Temperature Zone 4	210	${}^{\circ }$C

**Table 2:** Irradiation dose of TPE-E and V-TPE-E.

Required Irradiation Dose (kGy)	Real Surface Irradiation Dose (kGy)
0	0.0
33	38.7
66	77.4
99	116.1
132	154.8
165	193.5
198	232.2

**Table 3:** Gel Content.

Radiation Dose	0 kGy	33 kGy	66 kGy	99 kGy	132 kGy	165 kGy	198 kGy
TPE-E	0%	0%	0%	57%	69%	69%	74%
V-TPE-E	0%	0%	78%	79%	87%	92%	93%
